# Reduction of TRAF3 by heterozygosity or aging impacts B cell function

**DOI:** 10.1073/pnas.2507217122

**Published:** 2025-08-07

**Authors:** Emma L. Hornick, Kyp Oxley, Nathaniel Wieting, Emma Treco, Bruce S. Hostager, Gail A. Bishop

**Affiliations:** ^a^Department of Microbiology and Immunology, University of Iowa, Iowa City, IA 52242; ^b^Holden Comprehensive Cancer Center, University of Iowa, Iowa City, IA 52242; ^c^Veterans Affairs Medical Center, Iowa City, IA 52246

**Keywords:** B lymphocyte, cell signaling, TRAF

## Abstract

TNF receptor-associated factor 3 (TRAF3) is a signaling adaptor protein that is ubiquitously expressed but has highly distinct cell type–specific functions. TRAF3 plays critical roles in restraint of B lymphocyte activation, differentiation, and homeostatic survival. Consistent with such roles, loss-of-function mutations in *TRAF3* have long been found in various human B cell malignancies. Mice lacking TRAF3 specifically in B cells have autoimmune manifestations, lymphadenopathy, and increased incidence of B cell lymphomas. More recently, human patients with germline *TRAF3* mutations leading to haploinsufficiency have been reported; the phenotypes of these patients show striking similarities with those of mice with TRAF3-deficient B cells. This raises the important knowledge gap of how relative quantity of TRAF3 protein regulates B cells. To address this question, we investigated the effect of decreased B cell TRAF3 using mice whose B cells are heterozygous for loss of *Traf3*. *Traf3*^+/−^ B cells displayed multiple functional abnormalities, to an extent intermediate between *Traf3^+/+^* and *Traf3^−/−^* B cells, indicating a striking dose–response of B cells to relative quantities of TRAF3. Additionally, B cell TRAF3 protein—but not transcript—was reduced in B cells from normal aged mice and humans, consistent with increased occurrence of both B cell hyperactivity and B cell malignancies in older populations. Treatment of aged mice with a proteasome inhibitor restored the level of B cell TRAF3, suggesting age-related chronic signaling through receptors that lead to TRAF3 degradation. Thus, relative levels of B cell TRAF3 protein have important biological impacts on B cell function.

## Significance

Tumor necrosis factor (TNF) receptor-associated factor 3 (TRAF3) is an adaptor protein with highly context-dependent functions. Humans with a mutant *TRAF3* allele have autoimmune disease, immunodeficiencies, and increased risk of B cell malignancies. Studies in B cell–specific knockout mice and TRAF3-deficient human B cell lines establishes that TRAF3 regulates B cell survival and activation. This study investigated impacts of decreased B cell TRAF3 due to heterozygosity and aging. Reduced B cell TRAF3 protein had important dose-dependent effects on B cell functions, highlighting TRAF3’s key role in proper regulation of B cell homeostasis, activation, and differentiation. Results also emphasize the potential impact of age-related TRAF3 decline on increased frequency of disorders of B lymphocytes in older adults.

Control of cellular behaviors requires appropriate responses to signals that cells receive from their microenvironment. Aberrancies in signaling and its regulation underlie virtually all cellular dysfunction, and precise regulation of cellular survival and activation pathways is critical to maintain health of organisms. Dysfunction of B lymphocytes is intricately linked to a spectrum of diseases, including those of autoimmunity and chronic inflammation, as well as the immune response to tumors, and malignancies of B cells themselves (1). One of the key players serving as a rheostat for multiple B cell signals and thus functions is the signaling adaptor protein TNF receptor-associated factor 3 (TRAF3) (2).

TRAF3 plays multiple roles in the regulation of immune cell functions, roles that vary by cell and receptor type (3). In B lymphocytes, TRAF3 inhibits homeostatic survival, and also restrains signaling through multiple receptors regulating B cell activation and differentiation (4–11). TRAF3-deficient B cells exhibit substantially enhanced survival, which in mice results in marked lymphoid organ enlargement, production of autoantibodies, and development of B cell lymphomas (BCL) with age (4). It was recently appreciated that in humans, some individuals have only one functional copy of the *TRAF3* gene due to single-allele loss-of-function (LOF) germline mutations. *TRAF3* haploinsufficiency in humans mimics many of the phenotypes observed in cell type–specific *Traf3*^−/−^ mouse strains, particularly B-*Traf3^−/−^* mice (4, 12). For example, humans with *TRAF3* haploinsufficiency -like B-*Traf3^−/−^* mice- exhibit increased incidence of B cell–driven autoimmune diseases and BCLs (12).

TRAF3 exerts its impact on homeostatic survival in the cytoplasmic and nuclear compartments. In the cytoplasm, it regulates nuclear factor kappa-light-chain-enhancer of activated B cells 2 (NF-κB2) activation and cellular abundance of the survival-associated proteins Pim2 and c-Myc (6–8). It also inhibits nuclear levels of the transcriptional regulator CREB, thereby restraining transcription of CREB targets (e.g., the antiapoptotic protein Mcl1) (7). Deficiency of B cell TRAF3 also impacts glucose metabolism and IL-6 receptor signaling (6–8). *TRAF3* genetic mutations or deletions are a common finding in B cell malignancies, but B cells can also become functionally deficient in TRAF3 protein via posttranslational mechanisms (13). Signaling through activating receptors CD40 and receptor for BAFF (BAFFR) induces a cascade resulting in TRAF3 polyubiquitination and proteasomal degradation, reducing levels of TRAF3 protein for hours or more after the signaling event (14–16).

The phenotypes of humans with *TRAF3* haploinsufficiency suggest that even a partial reduction in TRAF3 protein can significantly alter B cell function and contribute to disorders involving B cells (12, 17). We thus wished to investigate mechanistically the impact of partial loss of TRAF3 on specific TRAF3-regulated B cell signals and functions. In this study, we examined the effects of a B cell–specific loss of one *Traf3* allele on B cell phenotype and function, using our mouse model that closely reflects the phenotype of the human single-allele *TRAF3* loss. Our results show that B-*Traf3*^+/−^ mice have significantly lower TRAF3 protein and mRNA levels in B cells, correlating closely with increased spleen weight and splenic B cell numbers compared to wild-type (WT) littermate mice. B cells from B-*Traf3*^+/−^ mice also showed better survival in vitro, to an extent intermediate between that of WT and TRAF3^−/−^ B cells. Additionally, multiple previously defined TRAF3-regulated proteins and pathways were affected to a partial but clearly demonstrable degree in *Traf3*^+/−^ B cells, indicating a dose-dependent effect of TRAF3 protein levels upon regulatory pathways in B cells.

We also found that older mice and humans display reduced levels of B cell TRAF3 protein, but normal levels of its mRNA. We began to investigate potential mechanisms for this protein loss. Our initial findings suggest that chronic signaling through the BAFFR contributes to the age-related decrease in TRAF3 by promoting its proteasomal degradation. This has significant implications for the age-related increase in autoimmune disease and B cell malignancies observed in humans.

Overall, this study underscores the vital role of TRAF3 in proper regulation of B cell homeostasis, activation, and differentiation, and the potential impact of its age-related decline on the increased frequency of disorders of B lymphocytes in older adults.

## Results

### Effect of B Cell TRAF3 on B Cell Number and Phenotype.

**Fig. 1.**
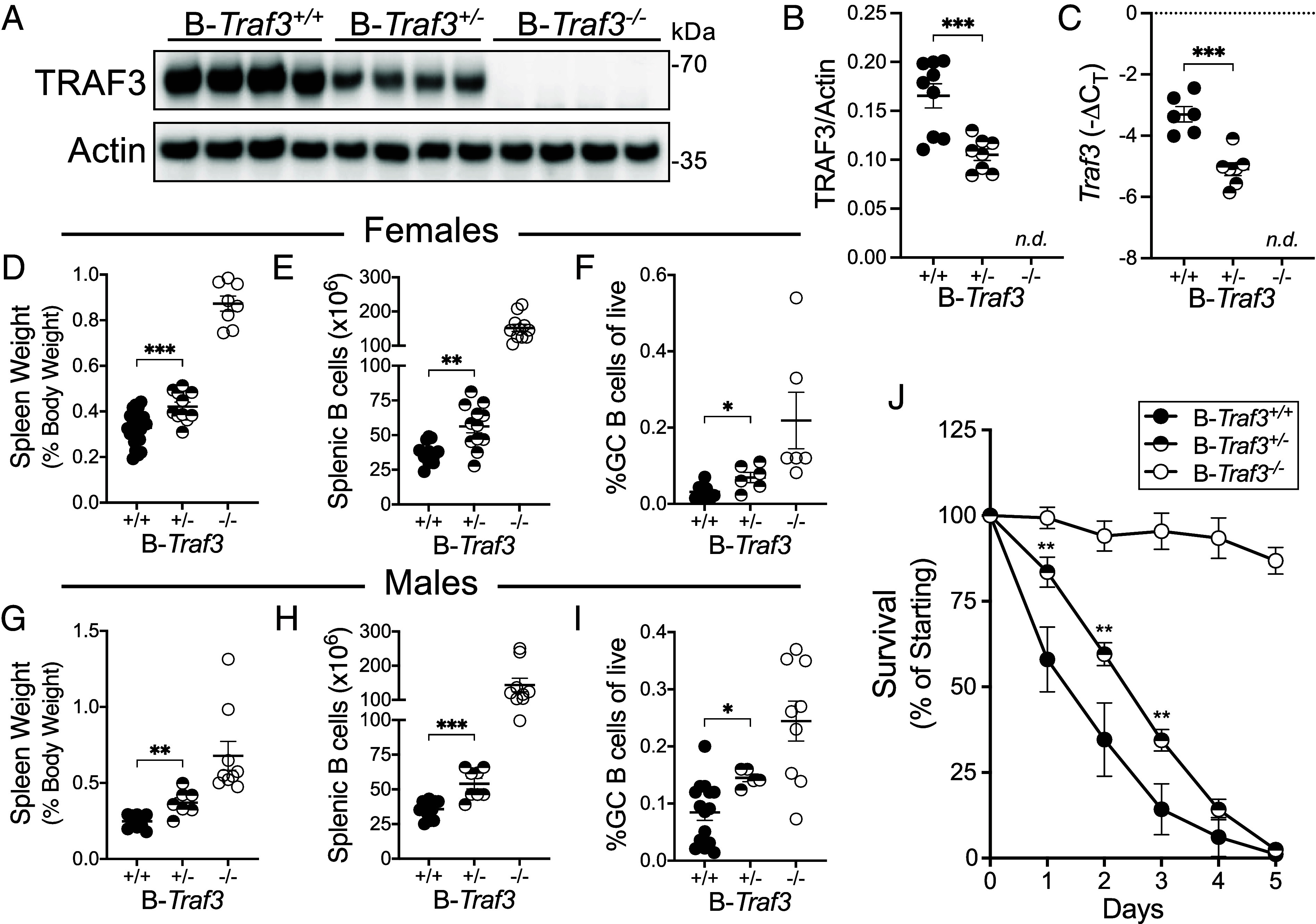
Effect of B cell TRAF3 abundance on B cell number and phenotype. (*A*) Representative western blot of TRAF3 protein in splenic B cells isolated from male and female mice of the indicated genotypes. Each lane is a sample from a single individual. (*B*) Quantification of splenic B cell TRAF3 protein from western blots including the blots shown in *A*. (*C*) Relative *Traf3* gene expression in freshly isolated splenic B cells from male and female mice, as determined by qRT-PCR. (*D*–*F*) Female mice; (*D*) Spleen weight at necropsy as a percentage of total body weight in mice of the indicated genotypes. (*E*) Splenic B cells were quantified after isolation from mice of the indicated genotypes. (*F*) Germinal Center (GC) B cells quantified by flow cytometry, presented as a percentage of live cells in spleens from mice of the indicated genotypes. (*G*–*I*) Male mice; (*G*) Same as *D*, for male mice. (*H*) Same as *E*, for male mice. (*I*) Same as *F*, for male mice. (*J*) Freshly isolated splenic B cells from male and female mice of the indicated genotypes were cultured in complete medium and counted daily for 7 d to determine survival. (*A* and *B*) Data are from two independent experiments with 8 to 9 mice per group. (*C*) Two independent experiments, 4 to 7 mice per group. (*D*–*I*) Three independent experiments, 5 to 14 mice per group. (*J*) Three independent experiments, 5 to 9 mice per group. Statistical significance was determined by the unpaired *t* test (*B*–*I*) or mixed-effects analysis (*J*). **P < 0.01, ***P < 0.001.

TRAF3 is an important negative regulator of many B cell activation and survival pathways (2). We were interested to determine whether a B cell–restricted loss of a single *Traf3* allele would affect B cell phenotype and function, and whether the amount of TRAF3 protein required for normal B cell functions would vary among different TRAF3-regulated pathways. We generated mice with B cells heterozygous for *Traf3* (B-*Traf3^+/−^*) and assessed the abundance of *Traf3* transcript and protein in their B cells. We found that B-*Traf3^+/−^* mice have a 40 to 50% decrease in the amount of TRAF3 protein and *Traf3* mRNA in their B cells (Fig. 1 *A*–*C*). B-*Traf3*^−/−^ mice have significantly elevated numbers of splenic B cells and have spontaneous germinal centers in the spleen (4), thus we next assessed spleen weight, splenic B cell number, and GC B cells in B-*Traf3^+/−^* mice. Spleen weight as a proportion of body weight, splenic B cell numbers, and the percent of B cells with a GC B cell phenotype (GL-7^+^CD95^+^) were significantly increased above those in WT littermates but remained lower than B-*Traf3*^−/−^ littermates (Fig. 1 *D*–*I*). There were similar trends for female (Fig. 1 *D*–*F*) and male mice of each of the three genotypes—WT, B-*Traf3^+/−^*, and B-*Traf3^−/−^* (Fig. 1 *G*–*I*).

TRAF3-deficient B cells exhibit enhanced homeostatic survival compared to WT B cells, leading to their accumulation in secondary lymphoid organs of B-*Traf3^−/−^* mice (4). Based on the elevation in splenic B cells in B-*Traf3^+/−^* mice, we predicted that B cells from B-*Traf3^+/−^* mice would survive longer in vitro than B cells from their WT littermates, but not as long as those in mice lacking all B cell TRAF3. Indeed, *Traf3^+/−^* primary mouse B cells exhibited significantly enhanced survival than WT for the first 3 d of in vitro culture, but both WT and *Traf3^+/−^* B cells were dead by day five (Fig. 1*H*). *Traf3^−/−^* primary mouse B cells showed superior survival compared to both WT and *Traf3*^+/−^ B cells from WT mice, remaining between 95 and 100% for at least 5 d in vitro (Fig. 1*H*).

### TRAF3 Protein Abundance Impacts TRAF3-Regulated Proteins and Pathways in B Cells.

**Fig. 2.**
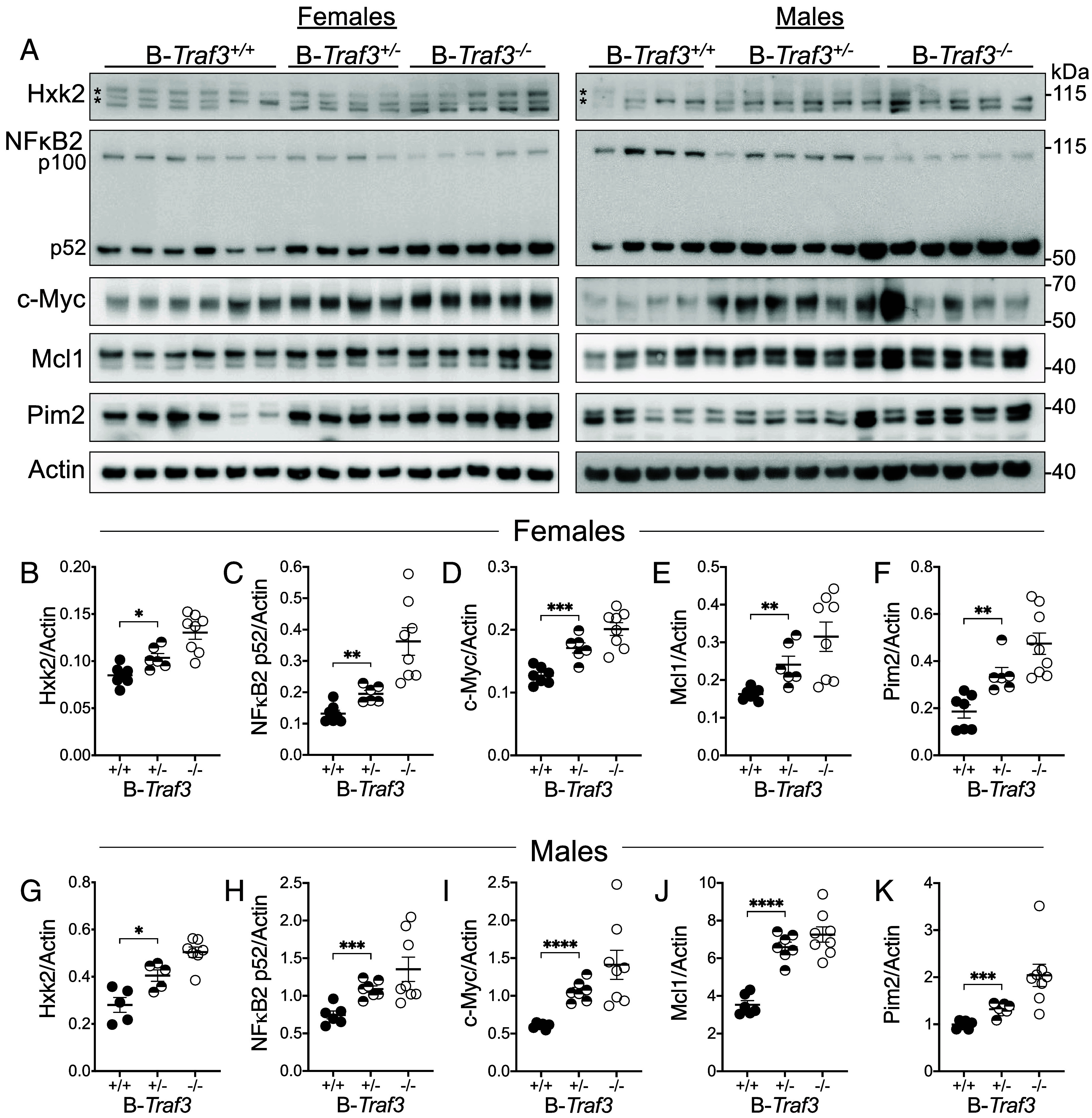
Impact of decreased B cell TRAF3 on TRAF3-regulated proteins. (*A*) Representative western blots of Hxk2 (* indicate nonspecific bands), NF-κB2, c-Myc, Mcl1, Pim2, and Actin in splenic B cells from female (*Left*) and male (*Right*) mice. Each lane is a sample from a single individual. (*B*–*F*) Quantification of the indicated proteins in splenic B cells from female mice, from western blots including those shown in *A*. (*G*–*K*) Same as *B*–*F*, for splenic B cells from male mice. Data are from three independent experiments, 6 to 9 mice per group. Statistical significance was determined by the unpaired *t* test (*B*–*K*). *P < 0.05, **P < 0.01, ***P < 0.001, ****P < 0.0001.

TRAF3 restrains B cell homeostatic survival through several different pathways and proteins, including antiapoptotic Bcl2 family member Mcl1, prosurvival kinase Pim2, glucose uptake and metabolism, and increased activation of the NFκB2 pathway (4, 7–9). We anticipated that one or more of these pathways in B cells from B-*Traf3^+/−^* mice might be unchanged from *Traf3*^+/+^ mice, affected similarly to *Traf3*^−/−^ mice, or intermediate between these genotypes, due to potential threshold effects. We also considered the possibility that the levels of TRAF3 required for normal regulation could differ between different functions and pathways. We quantified TRAF3-regulated proteins Hxk2, NFκB2 p52, c-Myc, Pim2, and Mcl1 in WT, *Traf3*^+/−^, and *Traf3^−/−^* B cells by western blotting. As with spleen weight and splenic B cell numbers (Fig. 1 *D*–*G*), though the relationships between genotypes were similar for each TRAF3-regulated protein, the total amounts in raw values from female mice (Fig. 1 *A*, *Left* and Fig. 1 *B*–*F*) and male mice (Fig. 1 *A*, *Right* and Fig. 1 *G*–*K*) differed enough that it was more accurate to consider them separately, rather than as pooled data. The glycolytic protein Hxk2, NFκB2 p52, Mcl1, c-Myc, and Pim2 were all significantly more abundant in *Traf3^+/−^* B cells compared to B cells from WT littermates (Fig. 2 *A*–*K*). Interestingly, the amount of these proteins was intermediate between WT and *Traf3^−/−^* B cells, suggesting a dose effect of cellular TRAF3 in its regulatory impacts upon its targets.

### B Cell IL-6 Receptor Signaling and Plasma Cell (PC) Differentiation in B-*Traf3^+/−^* Mice.

**Fig. 3.**
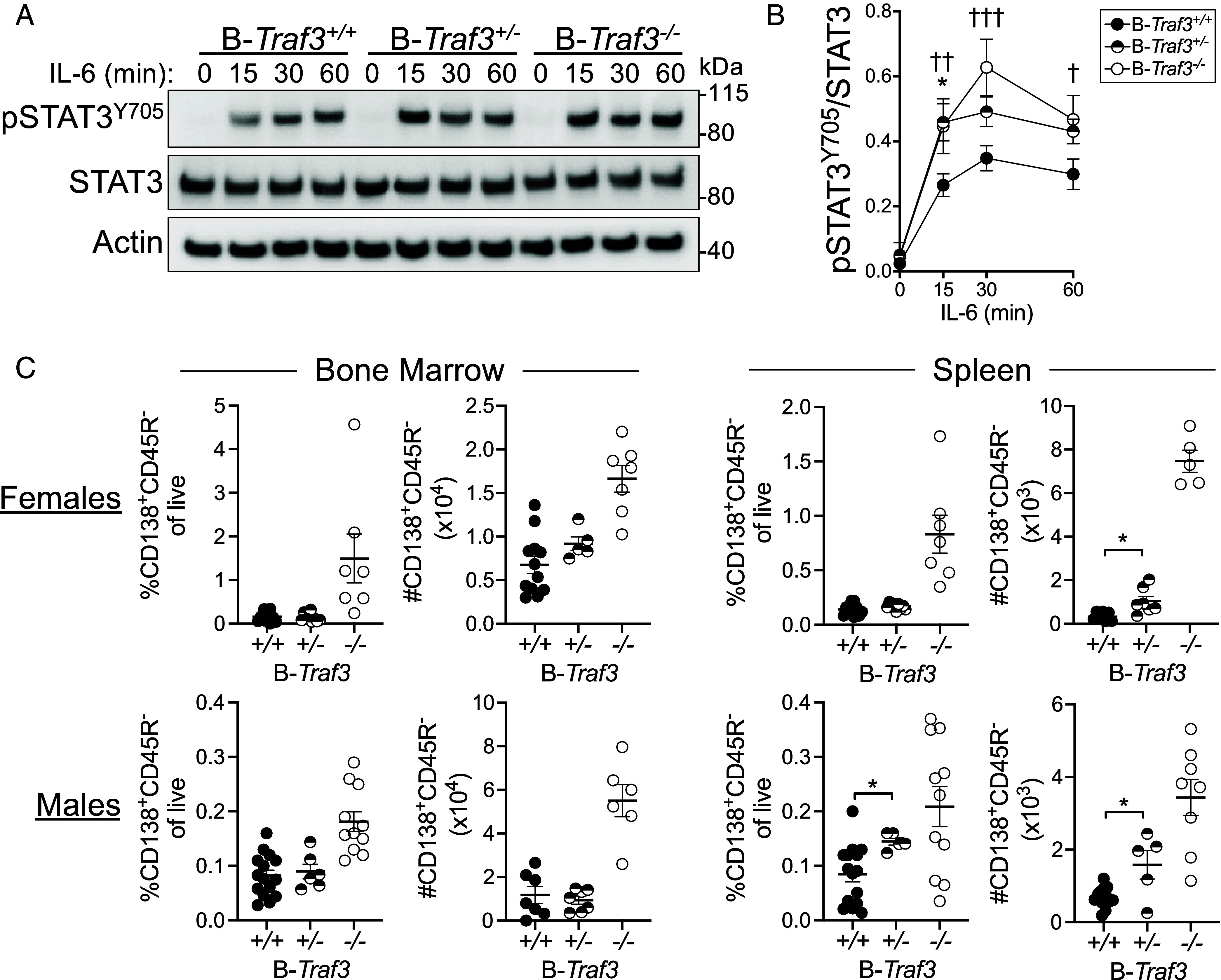
IL-6R signaling and PC differentiation in B-*Traf3^+/−^* mice. (*A*) Representative western blots of pSTAT3^Y705^, total STAT3, and Actin in freshly isolated splenic B cells of the indicated genotype stimulated with IL-6 for 15, 30, or 60 min, or left unstimulated. (*B*) Quantification of pSTAT3^Y705^ in IL-6-treated B cells from multiple experiments, including the blots in *A*. (*C*) PCs in bone marrow and spleen from male and female mice, quantified by flow cytometry. Data in *B* and *C* are from at least two independent experiments, 5 to 15 mice per group. Statistical significance was determined by repeated-measures ANOVA with Sidak’s multiple comparisons post hoc test (*B*), or unpaired *t* test (*C*). In *B*, † indicates comparisons between B-*Traf3^−/−^ and* B-*Traf3*^+/+^, * indicates comparisons between B-*Traf3^+/−^* and B-*Traf3^+/+^*. * or ^†^*P* < 0.05; ^††^*P* < 0.01, ^†††^*P* < 0.001.

IL-6 is an important B cell survival and PC differentiation signal (18–20). Work from our laboratory demonstrated that TRAF3 deficiency in B cells removes a crucial brake on IL-6R signaling, leading to a nearly twofold increase in PCs in B-*Traf3^−/−^* mice compared to WT littermate controls (6). We thus investigated whether IL-6R signaling was altered in *Traf3^+/−^* B cells. Consistent with our published results, *Traf3^−/−^* B cells had robustly increased phospho-STAT3 at Tyr705 compared to WT B cells at all timepoints following IL-6 stimulation (Fig. 3 *A* and *B*). *Traf3^+/−^* B cells had elevated pSTAT3 Y705 compared to WT B cells at all time points tested, although this increase reached statistical significance only at 15 min after IL-6 treatment (Fig. 3 *A* and *B*).

We next examined bone marrow PC accumulation in B-*Traf3*^+/−^ mice. In agreement with our previously published data, we found that all B-*Traf3^−/−^* mice had significantly increased populations of PCs (CD138^+^CD45R^−^) in spleen and bone marrow (Fig. 3*C*). B-*Traf3*^+/−^ mice had significantly increased numbers of PC in the spleen but not bone marrow compared to WT littermates (Fig. 3*C*). This suggests that relative amounts of TRAF3 may be more important for regulation of PC differentiation in different locations, which in turn may imply that IL-6R signaling exerts a greater regulatory role in the periphery than the bone marrow. Together, these data indicate that a reduction in B cell TRAF3 protein has important consequences for B cell survival and differentiation due to alterations in TRAF3-regulated signaling pathways.

### Effect of Aging on the Abundance of B Cell TRAF3.

**Fig. 4.**
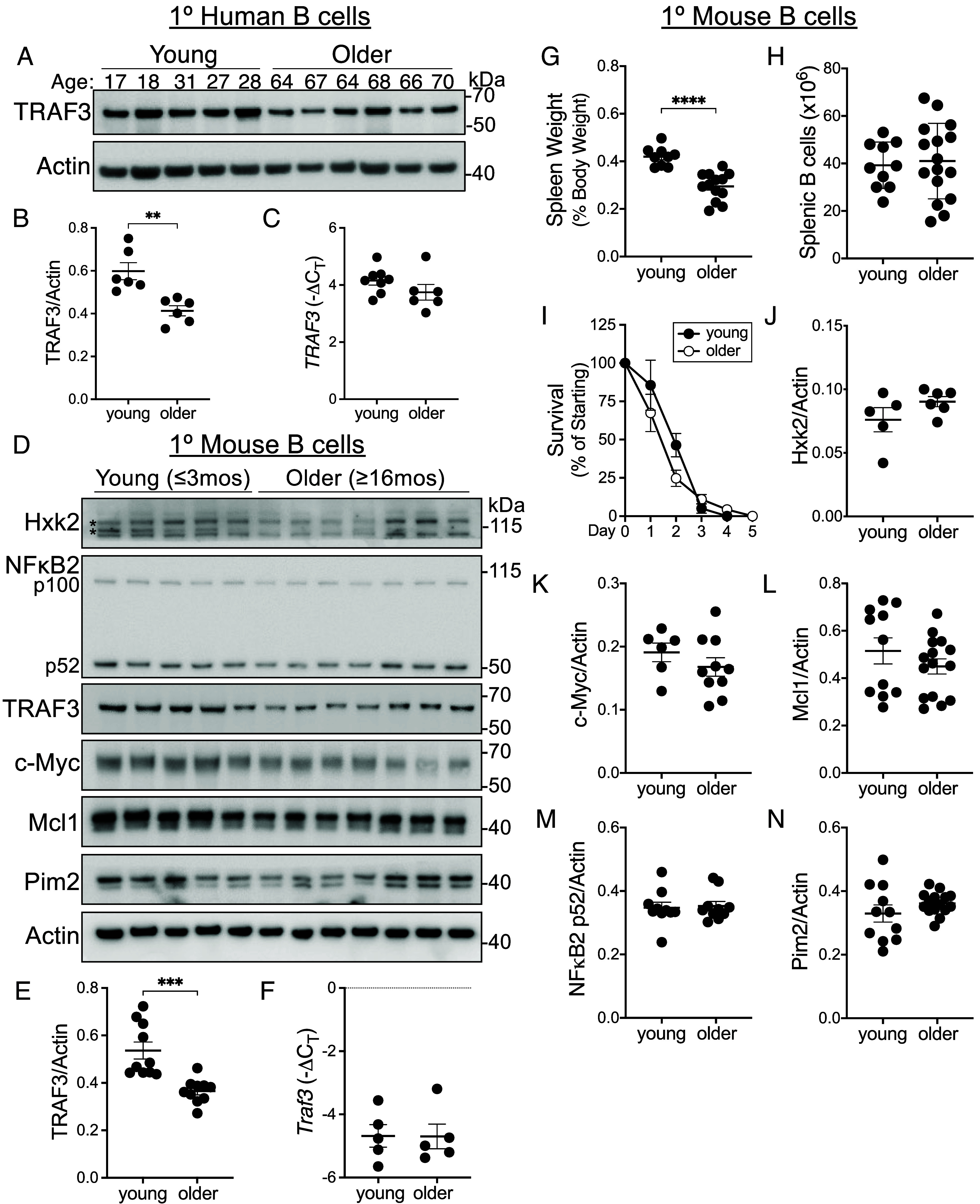
Effect of aging on mouse and human B cell TRAF3. (*A*) Representative western blot of TRAF3 protein in lysates of B cells isolated from the peripheral blood of healthy donors of the indicated ages. Each lane is a sample from a single individual. (*B*) Quantification of B cell TRAF3 protein from western blots including the blots shown in *A*. (*C*) Relative *TRAF3* gene expression in human B cells isolated from the blood of healthy young (17 to 31) or older (60 to 80) donors, determined by qRT-PCR. (*D*) Representative western blots of Hxk2 (* indicate nonspecific bands), NF-κB2, TRAF3, c-Myc, Mcl1, Pim2, and Actin in lysates of splenic B cells from young (≤3 mos) or older (≥16 mos) female mice. Each lane is a sample from a single individual. (*E*) Quantification of western blots of B cell TRAF3 protein, including the blots shown in *D*. (*F*) Relative *Traf3* gene expression in splenic B cells from young and older female mice. (*G*) Spleen weight at necropsy as a percentage of total body weight in young and older female mice. (*H*) Number of splenic B cells in young and older female mice. (*I*) Freshly isolated splenic B cells from young or older female mice were cultured in complete medium and counted daily for 5 d to determine survival. (*J*–*N*) Quantification of western blots for the indicated proteins in B cells, including those shown in *D*. (*B*) Data are from 10 to 11 individuals per group. (*C*) Data are from 6 to 10 individuals per group. (*D*–*N*) Data are from four independent experiments, 8 to 15 mice per group. Statistical significance was determined by the unpaired *t* test, **P < 0.01, ***P < 0.001, ****P < 0.0001.

TRAF3 protein is reduced in peripheral blood monocytes from healthy donors over 60 y of age compared to donors under 30 y of age (21). B cell function changes over the lifespan of humans and mice, with poorer humoral responses to infection and immunization and an increased incidence of both autoimmune disease and B cell–derived malignancies that corresponds to age (22). Given that activation and survival pathways are altered in B cells with reduced TRAF3 (12), we next determined whether B cells show a decline in TRAF3 with aging. Consistent with the findings in monocytes, we found that TRAF3 was decreased in B cells from healthy blood donors over the age of 64 compared to donors under the age of 32 (Fig. 4 *A* and *B*), but that *TRAF3* mRNA was not significantly decreased in B cells from older donors (Fig. 4*C*). We found a similar trend in B cells from young (≤3 mos) and older (≥16 mos) mice (Fig. 4 *D*–*F*).

Based on our findings in B-*Traf3^+/−^* mice, we predicted that the decline in aged mouse B cell TRAF3 would cause alterations in TRAF3-regulated pathways and processes. However, we did not find a clear effect of the reduced B cell TRAF3 upon the levels of several TRAF3-regulated prosurvival proteins in older mice (Fig. 4 *D*–*F*). Interestingly, spleens from older mice had a significantly reduced weight relative to young mice, but a similar number of splenic B cells (Fig. 4 *G* and *H*), suggesting that B cells represented an increased proportion of splenocytes in older mice. B cell survival was similar between older and young mice (Fig. 4*I*), as were TRAF3-regulated proteins Hxk2, NF-κB2, c-Myc, Mcl1, and Pim2 (Fig. 4 *D* and *J*–*N*). As aging alters many cellular functions in both immune and nonimmune cell types (23), such pleiotropic effects may overwhelm any discernible impact of the reduced TRAF3 in the sort of “snapshot” picture we can obtain in our experiments. In any case, the observed decrease in TRAF3 protein but not mRNA was intriguing. This may have important implications for the increased susceptibility of humans in the 6^th^ decade and beyond to the development of B cell malignancies, particularly BCL (23).

**Fig. 5.**
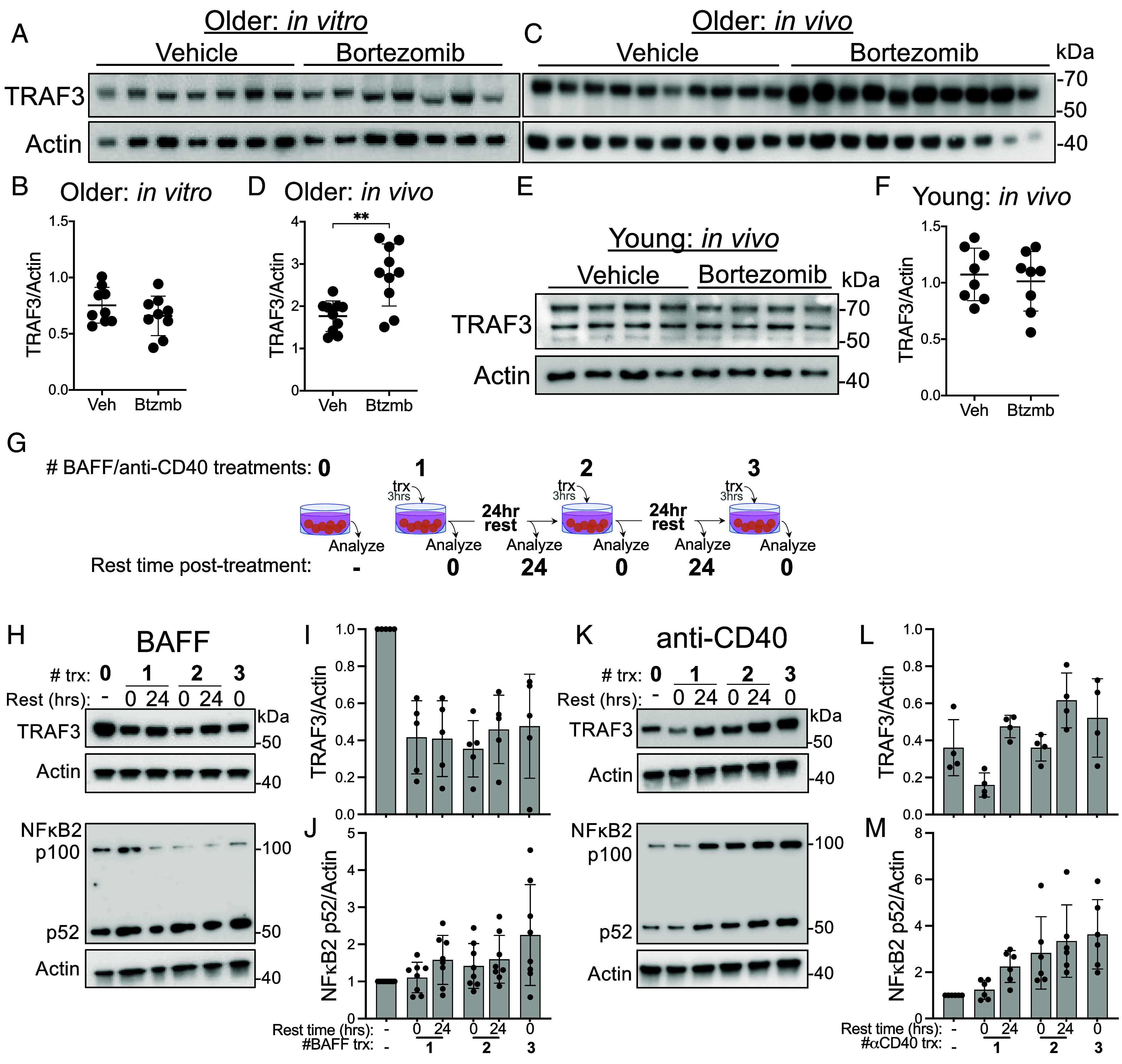
Chronic inflammatory signaling and B cell TRAF3 protein. (*A*) Representative western blot of TRAF3 in splenic B cells from older mice following culture in bortezomib or vehicle (dimethyl sulfoxide, DMSO) for 4 h at 37 °C, each lane is a sample from a single individual. (*B*) Quantification of blot shown in *A*. (*C*) Western blot of TRAF3 in splenic B cells from older mice treated with bortezomib 2 h prior to being killed and tissue harvest. Each lane is a sample from a single individual. (*D*) Quantification of western blot shown in *C*. (*E* and *F*) Same as *C* and *D*, using young mice instead of older mice. (*G*) Illustration of experimental design used to generate data *H*–*J*. (*H*) Representative western blots of TRAF3, NF-κB2, and Actin in mouse splenic B cells treated with BAFF as shown in *G*. (*I*) Quantification of western blots of TRAF3 in mouse splenic B cells following treatment with BAFF, including blots shown in *H*. (*J*) Quantification of western blots of NF-κB2 in mouse splenic B cells following treatment with BAFF, including blots shown in *H*. (*K*) Representative western blots of TRAF3, NF-κB2, and Actin in mouse splenic B cells treated with a stimulatory anti-CD40 antibody as shown in *G*. (*L*) Quantification of western blots of TRAF3 in mouse splenic B cells following treatment with anti-CD40, including blots shown in *K*. (*M*) Quantification of western blots of NF-κB2 in mouse splenic B cells following treatment with anti-CD40, including blots shown in *K*. (*B*) Data are from three independent experiments, nine mice per group. (*D*) Data are from three independent experiments, 10 mice per group. (*E*) Data are from two independent experiments, eight mice per group. (*I*) Data are from five independent experiments, five mice total. (*J*) Data are from eight individual experiments, eight mice total.

We next investigated the potential reason for decreased TRAF3 protein in B cells from older mice. Proteasome inhibition increases the amount of cellular TRAF3 in peripheral blood monocytes from healthy donors over the age of 65 (24); thus, we tested whether proteasome inhibition would have a similar effect on B cells from aged mice. However, in vitro proteasome inhibition with bortezomib did not alter the level of TRAF3 protein in B cells from older mice (Fig. 5 *A* and *B*). We reasoned that this could be due to the removal of the B cells from important signals in their in vivo environment. To capture the impact of those signals, we treated mice directly with bortezomib, then assessed the level of B cell TRAF3 protein. Consistent with this prediction, in vivo treatment with bortezomib for only 2 h was sufficient to increase the level of B cell TRAF3 in older mice, but not young mice (Fig. 5 *C*–*F*).

An increase in chronic, low-grade inflammation is a well-established aspect of aging that has profound effects on immune cell activation status and function (25). We therefore hypothesized that the decrease in B cell TRAF3 protein in older mice could be, at least in part, a result of chronic activation of inflammatory signaling pathways that induce proteasomal degradation of TRAF3. Upon engagement of their ligands, CD40 and BAFFR induce TRAF3 degradation (14–16). We examined the duration and extent of TRAF3 degradation in primary mouse B cells treated with BAFF (Fig. 5*G*). The initial 3 h treatment with BAFF reduced TRAF3 protein to about 50% of the amount in unstimulated cells (Fig. 5 *H* and *I*), a reduction that remained steady through 72 h after the initial stimulation (Fig. 5 *H* and *I*). CD40 stimulation, although also known to induce degradation of both TRAFs 2 and 3, had a very different impact: TRAF3 decreased after an initial anti-CD40 treatment as expected, but rebounded to greater than its starting amount by 24 h after the initial treatment (Fig. 5 *K* and *L*). Subsequent CD40 stimulation showed a similar pattern: TRAF3 protein decreased after the 3 h CD40 stimulation but recovered or exceeded its prestimulation level by 24 h after treatment (Fig. 5 *K* and *L*). The reduction in cellular TRAF3 induced by CD40 and BAFFR signaling activates the NFκB2 pathway through stabilization of NFκB-inducing kinase (26). We quantified p100 processing in primary mouse B cells that received repeated BAFF or CD40 stimulations. Levels of p52 relative to actin increased with additional stimulations (Fig. 5 *J* and *M*). This result suggests that while both BAFFR and CD40 can induce TRAF3 degradation, CD40 induces additional pathways that allow TRAF3 protein production to recover more rapidly than it does after BAFFR activation. However, the initial decrease in TRAF3 may well suffice to initiate activation of downstream NFκB2.

## Discussion

When our laboratory first developed the B cell–specific TRAF3 knockout mouse (B-*Traf3^−/−^*) (4), we noted that mice heterozygous for B cell–specific TRAF3 knockout (B-*Traf3^+/−^*) had enlarged spleens relative to WT mice, but remained significantly smaller than the spleens of B-*Traf3*^−/−^ mice. This hinted that loss of only one copy of *Traf3* might be sufficient to affect B cell survival and function, but until recently, there was very little information about the biological impact of a chronic partial loss of TRAF3. Subsequently, three independent reports of human patients with one normal and one LOF mutant TRAF3 allele (12, 17, 27) have underscored the significant impact of reduced TRAF3 upon immune cell function. Indeed, many of the clinical characteristics of these human patients overlap with the B-*Traf3^−/−^* mouse model, despite the remaining normal *TRAF3* allele in the cells of these patients (12, 17, 27). The phenotypic overlap suggests a prominent role for TRAF3-deficient B cells in the immune dysregulation that characterizes *TRAF3* haploinsufficiency in humans, though it is important to note that other cell types also require TRAF3 for optimal function and their dysfunction is certainly a contributing factor to disease in these patients (12). The work described in this manuscript expands our understanding of the dose dependence of TRAF3-regulated pathways in B cells and offers insight into the kinetics of TRAF3 recovery following stimuli that induce TRAF3 degradation.

We anticipated three possible types of effect of a partial loss of TRAF3 in *Traf3^+/−^* B cells: 1) a similar phenotype to WT B cells, 2) a phenotype intermediate between WT and *Traf3^−/−^* B cells, or 3) a similar phenotype to *Traf3^−/−^* B cells. We considered the possibility that there could be different impacts upon distinct TRAF3-regulated functions and pathways. However, for all the parameters we measured in this paper, the *Traf3^+/−^*B cells displayed an intermediate phenotype (Figs. 1–3). This result is internally consistent given the interrelated nature of TRAF3-regulated processes. For example, TRAF3 restrains homeostatic survival (28), so the increase in TRAF3-regulated proteins that promote B cell homeostatic survival (Mcl1, c-Myc, Pim2, and NFκB2, Fig. 2) (7, 9, 26) relative to WT B cells is the most likely explanation for increased in vitro survival of *Traf3^+/−^* B cells (Fig. 1). Similarly, increased survival predicts a greater accumulation of splenic B cells, leading to increased spleen weight (Fig. 1). Importantly, our results indicate that TRAF3 biological effects in B cells are mainly dose-dependent, rather than showing a threshold effect in controlling B cell signals and functions. These results thus clearly fill a major knowledge gap addressed by this study.

Although many of the TRAF3-regulated processes examined here were clearly and markedly affected by loss of a single copy of *Traf3*, IL-6R-mediated STAT3 activation was minimally affected (Fig. 3). pSTAT3 Y705 was significantly elevated in *Traf3^+/−^* B cells compared to WT B cells only at 15 min of IL-6 treatment, though this modification did appear to be trending toward significant increases at other time points. The increase in PCs, a parameter dependent upon elevated IL-6 (6) was also subtle, though statistically significant only in the spleen, whereas our previous work shows significant increases in both the spleen and bone marrow (6). Many signals are important for differentiation and survival of long-lived PCs (29); the IL-6-deficient mouse shows a mostly normal PC compartment (30, 31). The B cell antigen receptor (BCR) provides a crucial signal for B cell differentiation into antibody-secreting cells and PCs. BCR signaling is not affected by loss of a single copy of *Traf3*, but is enhanced in completely TRAF3-deficient B cells (11). The combined effect of enhanced BCR and IL-6R signaling in B-*Traf3^−/−^* mice may be driving the increased accumulation of long-lived PCs, whereas a slight enhancement in IL-6R signaling alone may be sufficient to initiate but not sustain the differentiation of long-lived PCs in B-*Traf3^+/−^* mice.

We found that TRAF3 protein was decreased in peripheral blood B cells from healthy human donors over age 65, and in splenic B cells from mice over age 16 mos (Fig. 4). Interestingly, only TRAF3 protein was decreased, but TRAF3 mRNA level was not affected in either mouse or human B cells (Fig. 4). In contrast to *Traf3*^+/−^ B cells, B cells from our cohort of aged mice did not have consistently altered levels of TRAF3-regulated proteins or in vitro survival (Fig. 4). The reduction in aged B cell TRAF3 was proteasome dependent, as treatment of aged but not young mice with the proteasome inhibitor bortezomib increased TRAF3 protein in B cells isolated from treated mice (Fig. 5). Together, our results revealed that, consistent with a previous study showing that TRAF3 protein level can be reduced by intracellular sequestration without genetic changes (32), posttranslational processes such as receptor-induced degradation resulted in chronically reduced TRAF3 protein in aged B cells.

It is interesting that despite the consistent decrease in B cell TRAF3 in older mice, we did not see a clear change in abundance of certain B cell TRAF3-regulated proteins, unlike in the B-*Traf3*^+/−^ mice. One possible explanation is related to the time required for reduced TRAF3 protein to have a quantifiable effect on B cell physiology. B cell TRAF3 is consistently decreased by 16 to 18 mos of age (Fig. 4). At that age, mice often have other age-related conditions that may affect responsiveness or activation state of immune cells and obscure a TRAF3-specific effect. Furthermore, we carefully examined aged mice at the time of tissue harvest and excluded any with signs of B cell malignancy, possibly excluding some of the subjects whose B cells were showing the effects of decreased TRAF3. Indeed, B-*Traf3^−/−^* mice have an increased incidence of BCLs beginning at around 9 mo of age (33).

We were encouraged to find that the age-related reduction in B cell TRAF3 was reversed by proteasome inhibition, which is consistent with the reported proteasome-dependent TRAF3 decrease in monocytes from older healthy human blood donors (21). However, in contrast to that report, TRAF3 protein levels were not restored when B cells were cultured with proteasome inhibitor in vitro (Fig. 5). This suggested that a signal present in vivo was required for the sustained reduction in B cell TRAF3. Signaling through CD40 or BAFFR leads to TRAF3 degradation in B cells, and chronic signaling through these receptors could contribute to sustained proteasomal degradation of TRAF3 protein in B cells from aged individuals (14–16, 34, 35). Sustained TRAF3 degradation would also be expected in patients with gain-of-function mutations in CD40 or BAFFR. Very few nonsynonymous mutations in the coding region of CD40 have been reported, suggesting that the functional WT sequence is critical for viability. However, increased expression of CD40 ligand (CD154) leading to more CD40 activation is associated with autoimmune diseases including SLE and Sjögren’s disease, both of which also affect patients with *TRAF3* haploinsufficiency (12, 36–38). Germline mutations in the coding region of BAFFR are also rare, though SNPs increasing serum BAFF levels have been associated with both an enhanced risk for developing non-Hodgkin’s Lymphoma and autoimmune diseases. At least one BAFFR mutation that enhances signaling and TRAF binding has been described in B lymphoma cells from multiple individuals (39).

Our studies revealed an important difference between the downstream effects of signaling via CD40 vs. BAFFR on TRAF3 protein level. Repeated CD40 stimulation was effective at inducing short-term TRAF3 degradation but led to an increase in B cell TRAF3 protein over time (Fig. 5). In contrast, BAFFR stimulation decreased TRAF3, and TRAF3 recovery took longer than after CD40 stimulation, and did not exceed the starting amount of TRAF3. Despite differences in the long-term effect on TRAF3 protein, both CD40 and BAFFR stimulation activated NFκB2 (Fig. 5), consistent with a prior study from our laboratory examining the relationship between TRAF3 degradation and NFκB2 activation (40).

Results presented here demonstrate a clear impact of partial TRAF3 reduction on a variety of important B cell signaling pathways and biology, and reveal that this impact is TRAF3 dose-dependent in its magnitude. Further, we report an interesting finding of reduced TRAF3 protein with normal *TRAF3* gene expression in aged B cells, that may contribute to the increased propensity for humans to develop B cell malignancies as they age (4, 12, 17, 33). Further exploration of these findings in both aging and in humans with *TRAF3* haploinsufficiency is warranted.

## Materials and Methods

### Mice.

Generation of mice with a B cell–specific loss of one (*Cd19*-Cre^+^*Traf3^flox/^*^+^, “B-*Traf3^+/−^*”) or both copies of *Traf3* (*Cd19*-Cre^+^*Traf3^flox/^*^flox^, “B-*Traf3^−/−^*”) was performed as previously described (4). For simplicity, *Cd19-*Cre^−^*Traf3^flox/flox^* are referred to as “WT” or “B-*Traf3*^+/+^”. Aged female C57BL/6 mice were acquired from the National Institute of Aging and used at 16 to 22 mo of age. Young female C57BL/6 mice (strain #000664) were acquired from The Jackson Laboratory and used at 2 to 3 mo of age. In some experiments, mice received an IP injection of 1 mg/mL bortezomib (Selleckchem, Houston, TX) or equivalent volume of vehicle (sterile normal saline, Bayer, Whippany, NJ) 90 min prior to being killed. All mice were housed in specific pathogen free facilities and used in accordance with the National Institute of Health guidelines under animal protocol #4052397 approved by the Animal Care and Use Committee at the University of Iowa.

### Human B Cells.

Human peripheral blood B cells were obtained from healthy anonymous donors at the DeGowin Blood Center at the University of Iowa. Under the blood center’s IRB-approved protocol #201402735, individuals who consent to donate whole blood or platelets also consent to having excess blood/blood derivatives used for research. Our laboratory received leukocyte reduction system (LRS) cones from healthy male and female platelet donors under age 33 or over age 63. Blood from LRS cones was diluted with Dulbecco’s Phosphate-Buffered Saline (DPBS, Gibco Life Technologies, Waltham, MA) and layered onto an equal volume of Ficoll-Paque Plus (Millipore Sigma, Burlington, MA), then centrifuged for 30 min at room temperature at 400× *g*. After centrifugation, the peripheral blood mononuclear cell (PBMC) layer was washed three times with DPBS containing Ca^2+^, Mg^2+^, and glucose (Gibco Life Technologies). B cells were enriched from PBMCs by negative selection using a human B cell enrichment kit (Stemcell Technologies, Vancouver, Canada).

### Cells and In Vitro Treatments.

Splenic mouse B cells were enriched using a negative selection kit (Stemcell Technologies, #19854). When splenic B cells were isolated from bortezomib-treated mice, 60 mM bortezomib was included in all isolation solutions. All mouse B cells were cultured in complete medium: RPMI 1640 (Gibco Life Technologies) containing 10% heat-inactivated fetal calf serum, 10 μM β-mercaptoethanol (Millipore Sigma), 2 mM L-Glutamine (Gibco), and 100 U/mL penicillin-streptomycin (Gibco). Quantification of mouse B cell survival in vitro was tracked as previously described (4). In vitro stimulation of mouse B cells with recombinant mouse IL-6 (PeproTech, Cranbury, NJ, 20 ng/mL), recombinant human BAFF (PeproTech, 100ng/mL), or anti-CD40 (clone G28.5, 10 μg/mL, BioXCell, Lebanon, NH) was performed at 37 °C in complete medium, followed by centrifugation and lysis for western blotting as described below. For proteasome inhibition in vitro, freshly isolated mouse B cells were incubated with 60nM bortezomib (Selleckchem) or equivalent concentration of vehicle (DMSO) for 4 h, followed by centrifugation and lysis.

### Western Blotting.

Cells were lysed in 1× Radio Immunoprecipitation Assay (Cell Signaling Technologies, Danvers, MA, #9806) Buffer supplemented with ethylenediaminetetraacetic acid-free cOmplete mini protease inhibitor cocktail (Roche Pharmaceuticals, Indianapolis, IN). Lysates were run on NuPAGE gels (Thermo Fisher Scientific, Waltham, MA) and transferred to polyvinylidene difluoride membranes with the XCell II blotting system (Thermo Fisher Scientific) according to manufacturer instructions. Nonspecific binding to membranes was blocked by incubating in 5% nonfat milk for 30 min at room temperature, followed immediately by incubation with primary antibody (1° Ab) overnight at 4 °C. The following 1° Abs were used for western blotting: Cell Signaling Technologies: TRAF3 (product #4729 and #33640), Hxk2 NFκB2 p100/p52 (#4882), c-Myc (#13987), Mcl1 (#94296), phospho-STAT3^Y705^ (#9145), STAT3 (#4904); Santa Cruz Biotechnology (Dallas, TX): β-Actin (#sc-47778), GAPDH (#sc-47724); Thermo Fisher Scientific: Pim2 (PA5-22315). Following 1° Ab incubation, membranes were washed and incubated with horseradish peroxidase (HRP)-tagged goat anti-mouse IgG (Jackson Immunoresearch Labs, West Grove, PA) or HRP-tagged goat anti-rabbit IgG (Jackson Immunoresearch Labs) for 2 h at room temperature. Blots were imaged using SuperSignal West Pico or Femto substrate (Thermo Fisher Scientific) and a low-light imaging system (LAS400, Fuji Medical Imaging, Lexington, MA). Densitometry of western blots was performed in ImageStudio Lite software (LI-COR Biosciences, Lincoln, NE) or MultiGauge Imaging software (Fuji Medical Imaging). Density of bands for proteins of interest was divided by density for loading control (Actin or GAPDH) from the same sample to normalize protein levels.

### Gene Expression Analysis.

RNA was extracted from mouse and human B cells using the RNeasy Plus Mini Kit (Qiagen, Germantown, MD) and quantified with a NanoDrop spectrophotometer (Thermo Fisher Scientific). cDNA synthesis was performed on equal amounts of starting RNA using the SuperScript III One-Step RT-PCR Supermix (Invitrogen) according to manufacturer instructions. Human and mouse *TRAF3* and *HPRT* (housekeeping) gene expression was measured using TaqMan probes (Applied Biosystems, Foster City, CA), TaqMan Fast Advanced Master Mix (Applied Biosystems), and a QuantStudio 3 RT-PCR machine (Applied Biosystems) with the following cycling conditions: 95 °C for 10min, 40 cycles of 15 s at 95 °C and 60 s at 60 °C (detection step). Data were analyzed using QuantStudio analysis software (Applied Biosystems).

### Flow Cytometry.

Single cell suspensions of mouse spleens and bone marrow from femurs and tibiae were stained for CD138 (clone 281-2, BioLegend, San Diego, CA), CD45R (clone RA2-6B3, BioLegend), CD95 (clone SA367A8, BioLegend), and/or GL-7 (clone GL7, BioLegend) in phosphate-buffered saline (PBS) containing 2% heat-inactivated fetal bovine serum, 2% rat serum (Jackson ImmunoResearch Labs), and Fc Receptor blocking mAb (anti-CD16/32, clone 2.4G2, Thermo Fisher Scientific) for 30 min at 4 °C. Cells were washed twice in PBS, fixed with BD Fluorescence-Activated Cell Sorting Lysis Buffer (Becton Dickinson, Franklin Lakes, NJ) for 10 min at room temperature, and resuspended in PBS. Data were collected on an LSR II (Becton Dickinson) flow cytometer and analyzed in FlowJo (Becton Dickinson).

### Statistical Analysis.

Data were graphed and the indicated statistical tests performed using GraphPad Prism software. Tests are specified in figure legends.

## Data Availability

All study data are included in the main text.
